# CLDN2 inhibits the metastasis of osteosarcoma cells via down-regulating the afadin/ERK signaling pathway

**DOI:** 10.1186/s12935-018-0662-4

**Published:** 2018-10-17

**Authors:** Xiaowei Zhang, Haiming Wang, Qian Li, Tao Li

**Affiliations:** 1 Center for Translational Medicine, Central Hospital of Zibo, Affiliated with Shandong University, Gong qingtuan Road 54Hao, Zibo, Shandong China; 2 Department of General Surgery, People’s Hospital of Linzi District, Affiliated with Binzhou Medical College, Zibo, Shandong China; 3 Department of Orthopedic Surgery, Central Hospital of Zibo, Affiliated with Shandong University, Zibo, Shandong China

**Keywords:** Afadin, Osteosarcoma, Claudin-2, Extracellular signal-regulated kinase, Metastasis, Tight junction

## Abstract

**Background:**

In an earlier study, we investigated the expression of tight junction protein claudins (CLDNs) in human osteosarcoma (OS) cells, and the CLDN2 was found to be down-regulated in primary tumor cells compared with normal osteoblast cells. Here, we sought to explore the effects of CLDN2 on the malignant phenotype of OS and the underlying molecular mechanisms.

**Methods:**

The expression patterns of CLDN2 and afadin in OS tissues and histologically non-neoplastic bone tissues were explored via immunohistochemistry and western blotting. CLDN2 expression levels in an OS cell line stably expressing CLDN2 and an osteoblast cell line with a CLDN2 knockout were confirmed by western blotting and immunofluorescence staining. The malignant phenotype of OS cells and osteoblast cells in vitro was assessed using a cell counting kit-8 assay, transwell assay and wound-healing experiment. Western blotting was utilized to detect the activation state of Ras/Raf/MEK/ERK pathway. Moreover, an RNA interference method were used to silence afadin in CLDN2-expressing OS cells.

**Results:**

Our research group found that CLDN2 and afadin was underexpressed in OS tissues, and the overexpression of CLDN2 significantly inhibited the migration abilities of OS cells. Genetic silencing of afadin in CLDN2-overexpressing OS cells promoted U2OS cell motility and activation of the Ras/Raf/MEK/ERK pathway.

**Conclusions:**

In this study, we confirmed that CLDN2 expression significantly inhibited the malignant phenotype of OS cells in vitro. Inhibition of the ERK pathway by afadin may be one of the mechanisms by which CLDN2 blocks the metastasis phenotype of OS cells.

## Background

The main reason for the poor prognosis of osteosarcoma (OS) is recurrence and metastasis, and the 5-year survival rate after recurrence and metastasis is less than 30% [1, 2]. At present, the issue of controlling the early metastasis of OS has become a bottleneck in the treatment of this disease [3]. Previous studies have shown that for various human malignancies, metastasis is accompanied by abnormalities in tight junction (TJ) structure and function [4, 5]. TJs are located at the very apex of cell junctions and both regulate cell adhesion and maintain cell polarity and permeability [6]. Recent research has shown that in addition to their functions in the maintenance of cell polarity and permeability, TJs are also reportedly involved in the transduction of intracellular/extracellular signals and the regulation of cell proliferation, differentiation, invasion and migration [7, 8]. Claudins (CLDNs) are the structural proteins that form TJs, and abnormalities in tight junction structure and function caused by a lack of CLDN expression on the cell membrane are required for tumor cell invasion and metastasis [9, 10]. Moreover, a growing number of studies in recent years have revealed that tumor cells frequently exhibit changes in the expression and localization of CLDNs [11]. For instance, it has been reported that CLDN1 is a metastasis suppressor for lung adenocarcinoma and that a lack of CLDN1 expression is associated with poor patient prognosis [12]. In addition, studies by Usami et al. indicated that reduced CLDN7 expression in head and neck cancer cells promoted cell invasion and migration [13]. These studies indicate that specific expression patterns of CLDNs in tumors have the potential to be used as molecular markers of malignancies.

Accumulating evidence suggests that the carboxyl termini of CLDNs, which are located in the cytoplasm, participate in signal transduction through their PDZ (PSD-95/Dlg/ZO-1 homology domain)-binding domains, which bind to cytoplasmic PDZ motif-containing proteins, including zonula occludens (ZOs) and afadin, among others [14–16]. The binding of CLDNs to these proteins constitutes a step in cellular signal transduction [17]. Of these binding partners, afadin is a core TJ protein that can bind to other TJ proteins in the cell membrane under certain physiological conditions and play an important role in maintaining TJ functions [18]. Furthermore, recent studies have shown that afadin can competitively inhibit the binding of rat sarcoma (Ras) protein to rapidly accelerated fibrosarcoma (Raf) protein, inhibiting the activation of mitogen-activated protein kinase kinase 1/2 (MEK1/2) and blocking activation of the extracellular signal-regulated kinase (ERK) signal pathway [19]. However, no prior reports have described either the role of CLDN2 on the malignancy phenotype of OS cells or whether the impact of CLDN2 is related to the afadin/ERK signaling pathway. In this study, we used molecular biological and other techniques to study the role and mechanisms of CLDN2 in OS cell metastasis and to identify novel targets for OS treatment and the control of early metastasis.

## Methods

### Cell culture

The human embryonic kidney cells 293 (HEK293T) cells, the human OS cell lines, U2OS and Saos2, and human fetal-osteoblast cell line, hFOB.1.19 were used in this study were purchased from Shanghai Cell Bank of the Chinese Academy of Sciences. These cell lines were maintained in Dulbecco’s modified Eagle’s medium supplemented with 10% fetal bovine serum (FBS) in a humidified incubator containing 5% CO_2_ at 37 °C.

### Plasmid construction and transfection

The pNSE-IRES-EGFP1**-**C1/CLDN2 (NM_001171095) was constructed and amplified by KeyGEN BioTECH Company. Five micrograms of each plasmid were transfected into cells using SuperFect Transfection Reagent (TaKaRa, Japan) in accordance with the protocol. G418 (Sigma, St. Louis, Missouri, USA)-resistant clones were expanded in culture as a monoclonal population. Cells transfected with an empty vector pNSE-IRES2-EGFP1-C1 (+) were used as a vector control.

### Realtime reverse transcript-polymerase chain reaction (RT-PCR)

Total RNA was extracted using a Perfect Pure RNA Cultured Cell Kit (ThermoFisher Scientific, Waltham, MA) in line with the manufacturer’s protocol. Real-time PCR reactions were carried out as previously pronounced [20]. Primers pairs of CLDNs and glyceraldehyde phosphate dehydrogenase (GAPDH) were as follows: CLDN1 forward (5′-GCCACAGCAAGGTATGGTAAC-3′) and reverse (5′-AGTAGGGCACCTCCCAGAAG-3′); CLDN2 forward (5′-TTCATCGGCAACAGCATCG-3′) and reverse (5′-GGTTATAGAAGTCCCGGATGA-3′); CLDN3 forward (5′-AGTGCAAGGTGTACGACTC-3′) and reverse (5′-AGTCCCGGATAATGGTGTTG-3′); CLDN4 forward (5′-TTGTCACCTCGCAGACCATC-3′) and reverse (5′-GCAGCGAGTCGTACACCTTG-3′); CLDN5 forward (5′-AACATCGTGACGGCGCAGACCA-3′) and reverse (5′-TCAGAGCCAGCACCGAGTCGTACA-3′); CLDN6 forward (5′-GGCAACAGCATCGTCGTGG-3′) and reverse (5′-GAAGTCCTGGATGATAGAGTGGGC-3′); CLDN7 forward (5′-TTTTCATCGTGGCAGGTCTT-3′) and reverse (5′-GGCCAAACTCATACTTAATGTTGG-3′); CLDN8 forward (5′-TCTGCAGTAGGA CATAGAAACCCCTAA-3′) and reverse (5′-CGTTTAGGGGTTTCTATGTCCTACTGC-3′); CLDN9 forward (5′-CTAGCACTAGTTTCGAAATGGCT TCGACCGGCTTAG-3′) and reverse (5′-TCTCGAGCTAGTCGACTCACACGTAGTCCC TCTTGTC-3′) and GAPDH forward (5′-AACGTGTCAGTCGTGGACCTG-3′) and reverse (5′-AGTGGGTGTCGCTGTFGAAGT-3′). The reverse transcript cDNA reaction products were subjected to quantitative real-time PCR using CTFX 96 Real-time system (Bio-Rad, Hercules, CA, USA) and SYBR green supermix (Bio-Rad, Hercules, CA, USA) according to the manufacturer’s protocol.

## Materials

Rabbit polyclonal antibodies against CLDN2 (ab107059) and anti-human β-actin (ab8226) were purchased from Abcam (Massachusetts, US). Rabbit anti-human phospho-ERK1/2 (#9251), rabbit anti-human ERK1/2 (#4695), rabbit anti-human ras (#14429), rabbit anti-human raf-1 (#2330), rabbit anti-human phospho-MEK1/2 (#2338), rabbit anti-human MEK1/2(#8727) and rabbit anti-human afadin (#13531) were purchased from Cell Signaling Technology (Boston, USA) and a streptavidin-peroxidase immunohistochemistry reagent kit was purchased from Maixin Biology (Fujian, China).

### Western blotting

The protein concentration of cell lysates was determined using a bicinchoninic acid (BCA) Protein Assay Kit (Pierce Chemical Co., Rockford, Illinois, USA). Then, 30 µg of total protein was separated via 10% SDS-PAGE and transferred onto nitrocellulose membranes (Millipore, Temecula, California, USA). Next, the membrane was blocked and investigated with rabbit anti-human phospho-ERK1/2 antibody, rabbit anti-human ERK1/2 antibody, rabbit anti-human p-MEK1/2 antibody, rabbit anti-human MEK1/2 antibody, rabbit anti-human CLDN2 antibody, mouse anti-human β-actin antibody, and rabbit anti-human afadin antibody at a 1:1000 dilution at 4 °C for 12 h. After three washes with phosphate-buffered saline (PBS), the membrane was incubated with horseradish peroxidase (HRP)-conjugated secondary antibody (Santa Cruz Biotechnologies, California, USA) at a 1:1000 dilution at room temperature (RT) for 30 min. Immunoreactive bands were detected using ECL western blotting reagents (GE, Fairfield, Connecticut, USA) and analyzed with Image Lab 6.0.1 Software from Bio-Rad Laboratories.

### Immunofluorescence method

Cells were washed thrice with phosphate-buffered saline (PBS), fixed with 4% paraformaldehyde for 10 min at RT, permeabilized with 0.1% Triton X-100 (Sigma-Aldrich; #9002-93-1) for 10 min at RT and blocked in 2% bovine serum albumin (Bote Biotechnological Corporation, Shandong, China) in PBS for 1 h at RT. Staining with primary antibodies was performed with rabbit anti-human CLDN2 and rabbit anti-human afadin antibodies, which was diluted in blocking solution (1:1000 dilution) for 30 min at RT. The cells were incubated with Alexa Fluor^®^647-conjugated anti-rabbit IgG antibody (ab150093, Santa Cruz Biotechnologies, California, USA) at a 1:1000 dilution. Images were taken using an Olympus IX81 microscope with an MT20/20 illumination system.

### Immunoprecipitation

Cells in the logarithmic growth phase were harvested and lysed in 600 μl of pre-chilled protein lysis buffer (4 °C) for 30 min. The lysate was centrifuged at 14,000 rpm at 4 °C for 20 min, after which the protein concentration was determined with a Beyotime protein assay kit. The protein supernatant was divided into two volumes. One 50-μl volume was mixed with 5× protein loading buffer, boiled in a water bath for 10 min, and then was stored at − 20 °C as the input control. The remaining volume of lysate was mixed with 30 µl of protein A/G agarose beads and 2 µg of a normal IgG antibody of the same species as the IgG used for immunoprecipitation. The mixture was rotated at 4 °C for 1 h and then centrifuged at 2500 rpm at 4 °C for 5 min, after which the supernatant was transferred to a new centrifuge tube. Next, 2 µg of the primary immunoprecipitation antibody (rabbit anti-human raf-1 antibody) was added to the total protein, and the mixture was adjusted to a final volume of 600 μl with protein lysis buffer. The mixture was slowly mixed overnight at 4 °C on a shaker. Forty microliters of adequately mixed protein A/G agarose beads was added to the mixture, which was slowly rotated at 4 °C for 90 min and then centrifuged at 3000 rpm at 4 °C for 10 min. Afterwards, the supernatant was removed, and the protein A/G agarose pellet was saved. Protein lysis buffer was used to wash the protein A/G agarose pellet five times, after which 25 μl of protein lysis buffer was added to resuspend the pellet followed by the addition of 5× loading buffer. The mixture was boiled for 5–10 min and used for sodium dodecyl sulfate-polyacrylamide gel electrophoresis (SDS-PAGE).

### Cell counting kit-8 assay

A cell proliferation curve was determined by the colorimetric water-soluble tetrazolium salt assay (Cell counting kit-8; Dojindo, Kumamoto, Japan) according to the manufacturer’s protocol. The cells were seeded into 96-well plates in triplicate, and cell proliferation was recorded every 12 h for 4 days.

### Colony formation assays

The experimental method was the same as describe previously [21].

### Flow cytometry cell cycle analysis

The experimental method was the same as describe previously [21].

### Wound-healing assay

Cells were cultured in a monolayer at 70% confluence on gridded plastic dishes. Monolayers were wounded by scratching with a 100-μl pipette tip and subsequently washed 3 times with phosphate buffer saline. Images of the wound site were taken using a light microscope (E100, Nikon Instruments Inc, Japan) (magnification 200×) at the same location at 0, 12 h and 24 h, respectively.

### Transwell chamber method

The cells were grown in a monolayer at 90% convergence and were maintained in FBS-free medium for 12 h. Matrigel (BD Biosciences, cat. no. 356234) was added to the upper Boyden chamber (Millipore, Bedford, MA) in 24-well plates and the plates were maintained in a cell incubator at 37 °C for 15 min. Then, medium containing chemotactic factors, which had been collected from the cell culture, was added to the 24-well plate. The cells were supplemented with Matrigel and cultured in a cell incubator at 37 °C for 6 h.

### Short hairpin RNA (shRNA) method

Frozen glycerol bacterial stocks containing pGCSIL-scramble, pGCSIL-CLDN2-RNAi and pGCSIL-afadin-RNAi frozen glycerol bacterial stocks were purchased from Nanjing KeyGen Biotech Co, Ltd. HEK 293T cells (0.2 × 10^7^) were seeded and maintained for 24 h to achieve 70–80% confluence in 6-well dishes (Costar, Cam- bridge, MA). The plasmids, including 10 μg pGCSIL-afadin-RNAi, pGCSIL-CLDN2-RNAi or pGCSIL-scramble, 5 μg packaging vector pHelper 1.0 and 5 μg vesicular stomatitis virus glycoprotein (VSVG) expression plasmid vector, were supplemented with Opti-MEM and 1.0 ml. A total of 50 μl of Lipofectamine was added to 950 μl FBS-free medium. These two mixtures were mixed and added to the cells. Lentiviral particles were harvested 48 h after transfection, and the viral titer was determined by counting green fluorescent protein (GFP)-expressing cells under fluorescence microscopy (Nikon Diaphot 300^®^) with filters 96 h after transfection.

### Patients and tissue samples

Biopsies were gathered from 27 patients with a pathologically confirmed diagnosis of OS who received treatment at the Qilu Hospital of Shandong University between June 2007 and May 2012. The patients were chosen based on the following criteria: no history of radiotherapy, no history of chemotherapy, and no prior malignant disease. The grade and classification of OS patients were referred to the American Joint Committee on Cancer (AJCC) tumor node metastasis (TNM) staging system. 3 of the biopsies were taken from the tibia, 3 from the humerus, 16 from the femur, 2 from the fibula, and 1 each from the forearm, the hand, and the pelvis. 21 of the cases also exhibited pulmonary metastasis. Bone tissue that was identified as histologically non-neoplastic was also obtained from 10 patients with osteoarthritis who were treated at the Qilu Hospital of Shandong University between October 2006 and September 2011. There were 6 men and 4 women with an average age of 47 years.

### Immunohistochemistry

Immunohistochemistry was used to detect the expression levels of CLDN2 and afadin in paraffin-embedded biopsy specimens from 27 patients who had a diagnosed primary OS and had not undergone chemotherapy or radiotherapy before biopsy. The experimental method was the same as described previously [21], and the antibodies used were rabbit anti-human CLDN2 and afadin antibody. Negative control slides were incubated with isotype antibodies at same dilution with primary anti-human CLDN2 and afadin antibody. The expression levels of CLDN2 and afadin located at the cell membrane and cytoplasm were taken as positive. The staining and scoring of the CLDN2 and afadin protein expression levels were classified semi-quantitatively based on the total combined scores of the percentage of positively stained tumor cells together with the staining intensity as previously described [22]. The final score of the protein expression was defined as ‘low’ if < 30% of tumor cells stained positive, and ‘high’ if > 30% of tumor cells stained positive. At least five different areas of the tumor were examined, and the mean of the results was used as the final expression score in each case.

### Statistical methods

All of the experiments were repeated three times, and all of the data are based on the mean ± SD of at least three experimental results. The Chi-square test/Chi-square goodness of fit test was used to analyze the correlations between CLDN2 and afadin expression and clinical pathological indicators. The results were analyzed by Student’s t-test, and *P *< 0.05 was considered to be statistically significant.

## Results

### Expressions of CLDNs family members in OS cell lines and osteoblasts

**Fig. 1:**
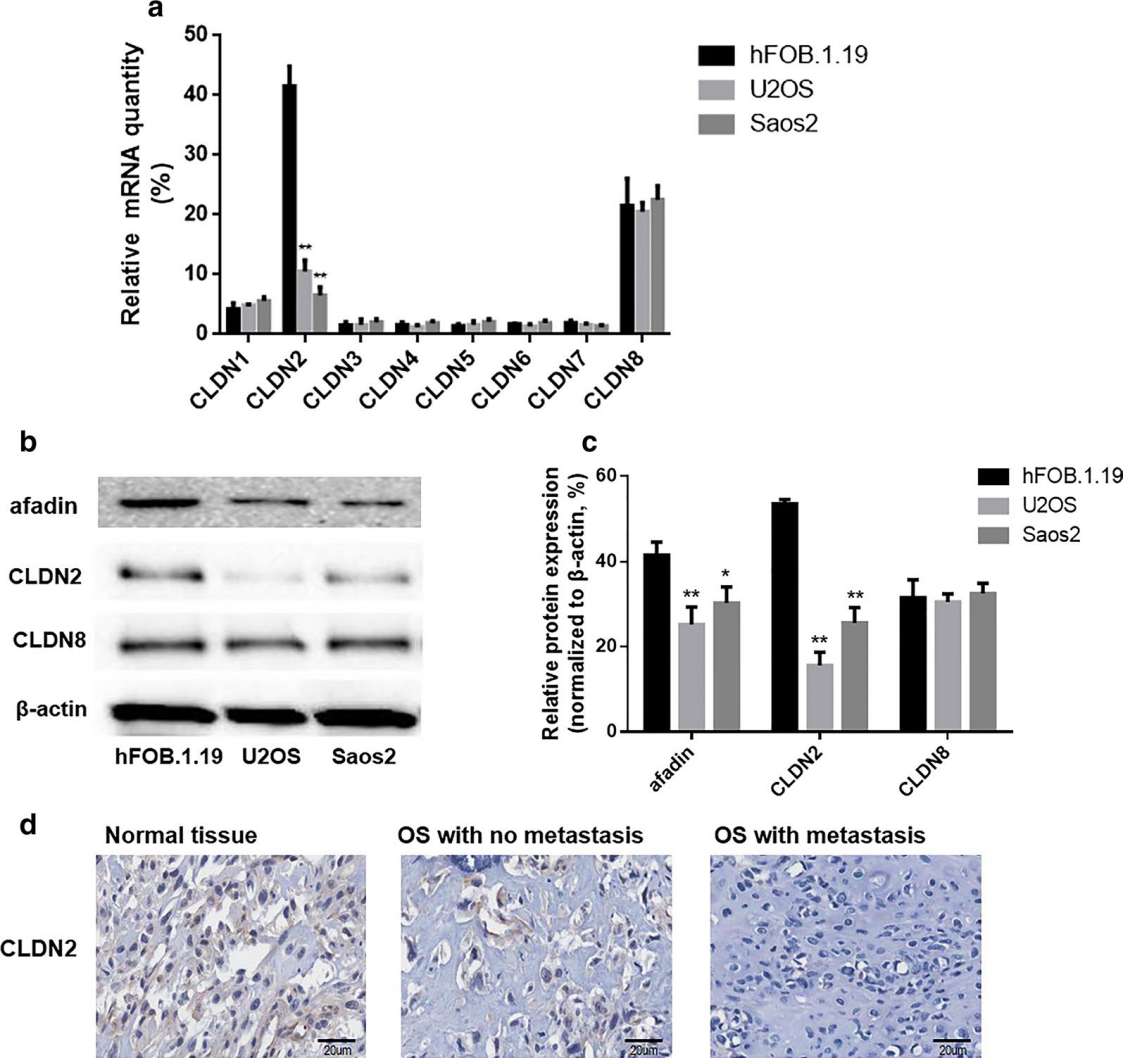
Expressions of CLDNs family members in OS cell lines and osteoblast line. **a** Detection of the expression of CLDNs family members in OS cell lines and osteoblast line by Realtime-PCR. **b** Detection of CLDN2 expression in OS cell lines and osteoblast line by Western blotting. **c** The corresponding statistical analysis of protein expression. **d** The protein expression of CLDN2 in OS tissues and normal bone tissues. Normalized with β-actin, the *P < 0.05, **P < 0.01 compared with the hFOB.1.19 group

Real-time quantitative PCR and western blotting were used to detect the expression of CLDNs family members in fetal-osteoblast cell line, hFOB.1.19 and OS cells U2OS, Saos2. The results showed that the mRNA and protein of CLDN1, CLDN3, CLDN4, CLDN5, CLDN6 and CLDN7 were not expressed in osteoblast cell lines and OS cell lines. Meanwhile, the mRNA and protein of CLDN8 was expressed in osteoblasts and OS cell lines, but there was no significant difference between the mRNA and protein expression of CLDN8 in osteoblasts and OS cell lines. Furthermore, the mRNA and protein of CLDN2 is expressed in low expression in OS cell lines and is high expressed in osteoblasts (Fig. 1a–c). It is suggested that the silence of CLDN2 expression may play a role in the occurrence of OS. Furthermore, the protein expression of afadin, a potential target gene of CLDN2, is also expressed in low expression in OS cell lines and is high expressed in osteoblasts.

Besides, CLDN2 expression was explored in 27 OS tissues and 10 histologically non-neoplastic bone tissues. As shown in Fig. 1d, the expression of CLDN2 in histologically non-neoplastic bone tissues and OS tissues is mainly located in the cytoplasm and membrane. High expression of CLDN2 was observed in 33.3% (9/27) of OS tissues and in 80.0% (8/10) of histologically non-neoplastic bone tissues (Table 1). These data revealed that the expression of CLDN2 was decreased in OS tissues compared with histologically non-neoplastic bone tissues (P = 0.0001) (Table 1). The relationship between CLDN2 and clinical pathological indicators was also analyzed, and it was found that the expression of CLDN2 was not associated with age (*P* = 0.871), gender (*P* = 1.000), stage (*P* = 0.726), or response to chemotherapy (*P* = 0.642) of OS patients. However, CLDN2 expression was associated with pulmonary metastasis (*P *= 0.001) (Table 1).

**Table 1:** Expression of CLDN2 and clinicopathological characteristics in OS patients

Item	N	CLDN2 (high)	CLDN2-negative (low)	*P*
Tumor tissue	27	9	18	< 0.001*
Normal	10	8	2	
Age (years)				
≤ 19	15	5	10	0.871
> 19	12	4	7	
Gender				
Male	16	5	11	1.000
Female	11	4	7	
Stage				
IA–IIA	10	3	7	0.726
IIB–III	17	6	11	
Response to chemotherapy				
Poor	9	2	7	0.642
Good	7	2	5	
NA (n = 8)	11			
Pulmonary metastasis				
+	21	6	15	< 0.001*
−	6	3	3	

*NA* not available

* Statistical significance was found with the Chi-square test/Chi-square goodness-of-fit test

### Stable transfection of OS cell line U2OS with CLDN2

**Fig. 2:**
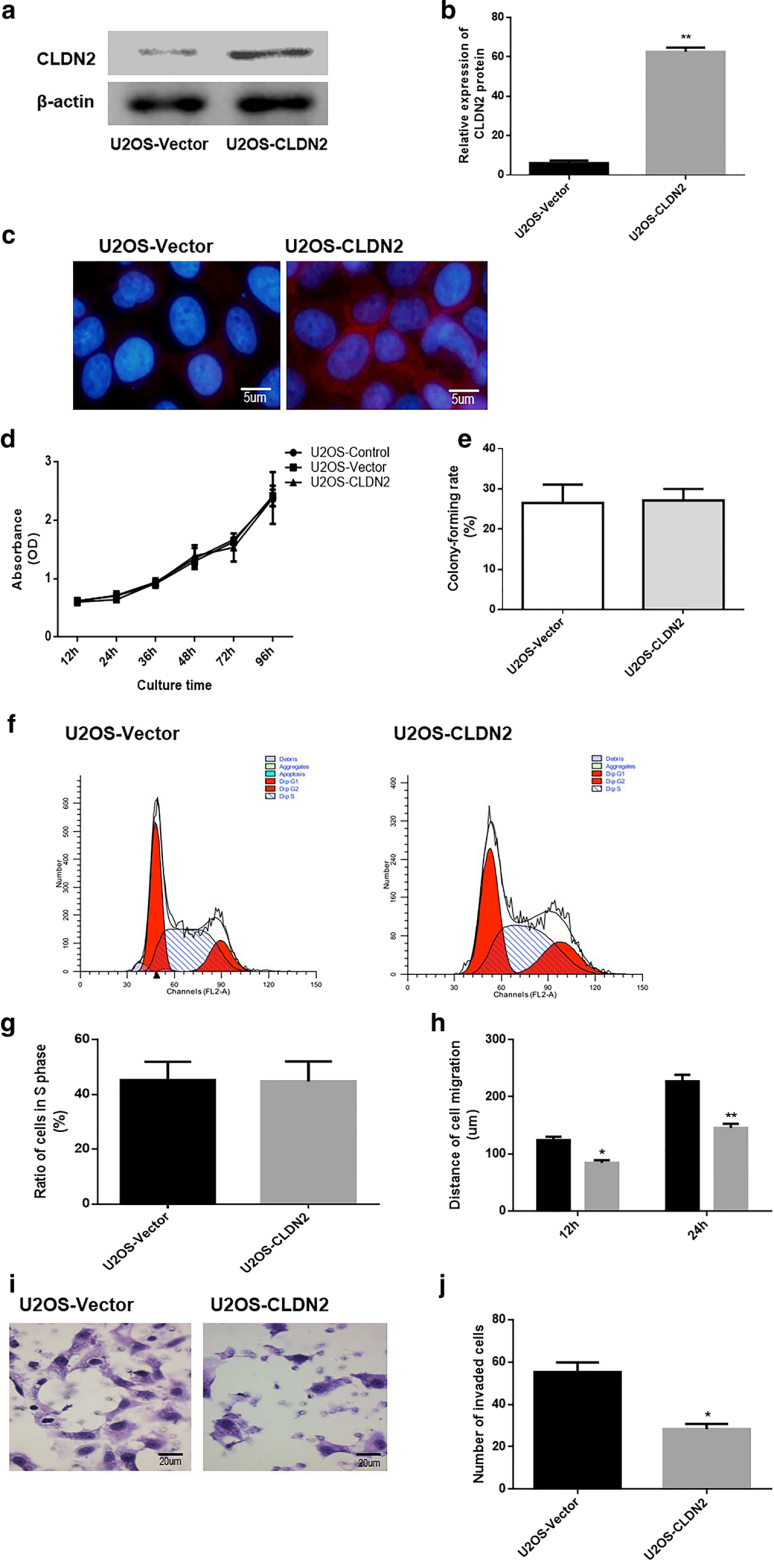
The impact of CLDN2 on the proliferation and metastasis ability of U2OS cells in vitro. **a** Detection of CLDN2 expression in the U2OS cells by Western blotting. **b** The corresponding statistical analysis of protein expression. **c** Detection of CLDN2 expression in the U2OS cells by immunofluorescence. **d** The growth curve of the U2OS cells was drawn by the CCK-8 method. **e** The abilities of U2OS cells to form colonies were detect by colony formation assay. **f** The cell cycle distribution of U2OS cells was analyzed using flow cytometry. **g** The percentage of cells with S phase DNA content from U2OS cells. **h** The transwell chamber method was utilized to detect the invasive ability of the U2OS cell line in vitro. **i** The wound healing assay was utilized to detect the migration ability of the U2OS cell line in vitro. *P < 0.05, **P < 0.01, compared with the control group compared with the vector group

In our presented work, CLDN2 was expressed at low level in Saos2 cells and undetectable in U2OS cells. Therefore, to examine the consequence of an increase in CLDN2 expression, we stably over-expressed CLDN2 in U2OS cells. A pNSE-IRES-EGFP1-C1/CLDN2 plasmid was used to transfect U2OS cells. After G418 screening, a mixer with ten monoclonal strains of U2OS cells transfected with a pNSE-IRES-EGFP1-C1/CLDN2 plasmid was obtained, which was termed U2OS-CLDN2. Western blotting and immunofluorescence were used to detect the expressions and localizations of CLDN2 in U2OS cells. The results showed that the protein expression levels of CLDN2 in the clonal U2OS cells were significantly higher (*P* = 0.0014) than those in the empty vector groups (Fig. 2a, b). Besides, the expression of CLDN2 was primarily localized on cell membrane and cytoplasm (Fig. 2c). The results demonstrated that U2OS cell line that stably expressed CLDN2 were successfully established.

### The impact of CLDN2 on the proliferation rate and metastasis ability of OS cell

The growth curve of U2OS cells was drawn by CCK-8 method. As it revealed in Fig. 2d, the data revealed that there was no significant difference in the proliferation rate between U2OS-CLDN2 cells and empty vector groups. We also determined the abilities of CLDN2-expressing cells to form colonies, in both 2D monolayer culture (Fig. 2e). there was no significant difference in the number of colonies formed between U2OS-CLDN2 cells and empty vector groups (*P *= 0.716). Moreover, we explored whether CLDN2 affect the cell cycle progression. The cell cycle distribution of U2OS cells was analyzed using flow cytometry. A representative histogram for each cell line is shown in Fig. 2f, g, and the percentage of cells with a S phase DNA content is plotted. The results revealed no differences in the percentage of U2OS cells in S phase (*P *= 0.647), between cells lines with and without CLDN2 expression, suggesting that CLDN2 has no effect on cell cycle progression.

A wound-healing experiment was also used to detect the impact of CLDN2 on the migration ability of OS cells (Fig. 2h). The results showed that at 12 h and 24 h, the migration distances of U2OS-CLDN2 (*P *= 0.036; *P *= 0.0021) was significantly shorter than those of the empty vector groups. To determine the impact of CLDN2 on cell metastasis, the transwell chamber method was used to detect the invasive ability of OS cells. Six hours after the U2OS cells were seeded, those cells that invaded under the membrane of the chamber were observed (Fig. 2i). The results showed that the numbers of invasive U2OS-CLDN2 (*P *= 0.016) cells were significantly lower than those for the empty vector groups (Fig. 2j). This result suggests that CLDN2 significantly inhibits the invasive ability of U2OS cells in vitro.

These results suggest that CLDN2 significantly inhibits the migration ability of OS cells in vitro.

### The impact of CLDN2 on the ERK signaling pathway of OS cells

**Fig. 3:**
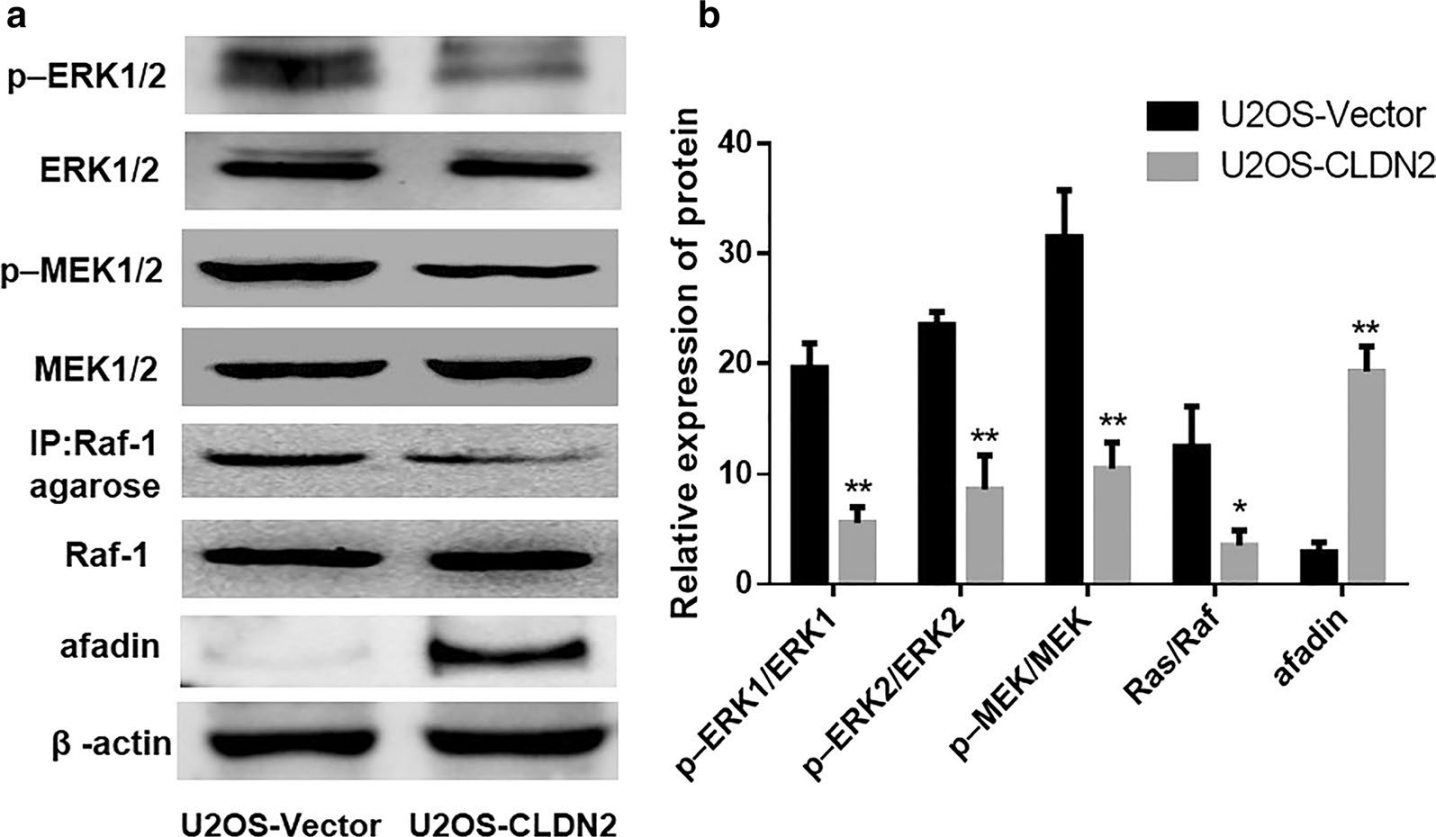
The impact of CLDN2 on the Ras/Raf/MEK/ERK signaling pathway of U2OS cells. **a** Western blotting was used to detect the activation of the Ras/Raf/MEK/ERK signaling pathway in U2OS cells. **b** The corresponding statistical analysis of the activation of the Ras/Raf/MEK/ERK signaling pathway. *P < 0.05, **P < 0.01 compared with the control group, compared with the vector group

Western blotting was used to detect the activation state of the Ras/Raf/MEK/ERK pathway. As it showed in Fig. 3a, b, after the overexpression of CLDN2, the ratio of phosphorylation ERK1/ERK1 (*P *= 0.0026) and phosphorylation ERK2/ERK2 (*P *= 0.0015) were significantly decreased in U2OS cells. Besides, the phosphorylation levels of MEK1/2 (*P *= 0.0023; *P *= 0.0017), an upstream regulator of ERK1/2, were also significantly decreased in the U2OS cells that overexpressed CLDN2. In addition, upstream Ras activity in the U2OS (*P *= 0.024) cells were significantly decreased accompanied by the overexpression of CLDN2. However, the expression of afadin which is a potential target gene of CLDN2 was significantly increased in U2OS-CLDN2 group (*P *= 0.0012). These data suggest that CLDN2 significantly inhibits the activation of the Ras/Raf/MEK/ERK signaling pathway.

### The impact of CLDN2 knockdown on the metastasis of fetal-osteoblast cell line

**Fig. 4:**
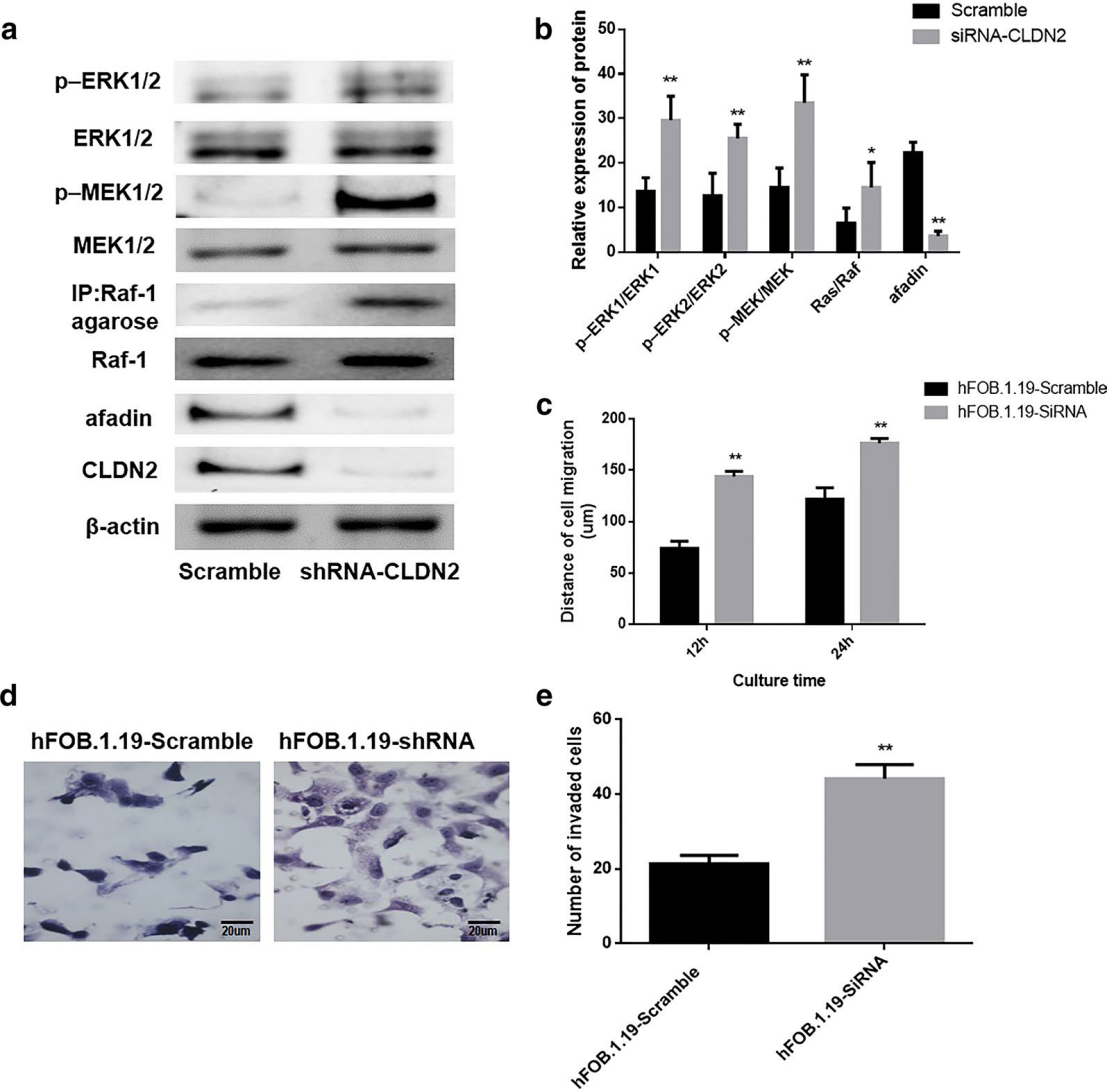
RNAi was utilized to silence CLDN2 expression in osteoblast line. **a** Western blotting was utilized to examine the effects of silencing CLDN2 and the activation of the Ras/Raf/MEK/ERK signaling pathway in the osteoblast line. **b** The corresponding statistical analysis of the activation of the Ras/Raf/MEK/ERK signaling pathway. **c** The wound healing assay was utilized to detect the migration ability of the osteoblast line in vitro. **d** The transwell chamber method was utilized to detect the impact of CLDN2 silencing on the invasive ability of cells in vitro. **e** The corresponding statistical analysis of invaded cells. **P < 0.01 compared with the scramble group

To determine the impact of CLDN2 on invasion and migration ability of osteoblast cell, we transfected fetal-osteoblast cell line, hFOB.1.19 with a pGCSIL-scramble plasmid and a pGCSIL-CLDN2-RNAi plasmid. The western blotting was used to analyze the expression of CLDN2 and changes in the activation state of Ras/Raf/MEK/ERK pathway in these cells, as it showed in Fig. 4a, b, the ratio of phosphorylation ERK1/2 (*P *= 0.0013; *P *= 0.0027) and phosphorylation MEK1/2 (*P *= 0.0026; *P *= 0.0003) were significantly increased in the hFOB.1.19 cells that silence CLDN2. In addition, upstream Ras activity in the hFOB.1.19 (*P *= 0.017) cells were significantly increased accompanied by the silence of CLDN2. However, the expression of afadin was significantly decreased in the hFOB.1.19 cells that silence CLDN2 (*P *= 0.0042).

The transwell chamber assay and wound-healing assay were used to analyze the effect of CLDN2 on the invasive and migration ability of the examined cells. The results showed that the migration distances of HFOB.1.19 cells in the CLDN2-RNAi group were significantly longer than those of the scramble group at 12 h and 24 h (*P *= 0.0008; *P *= 0.0001) (Fig. 4c). The numbers of invasive HFOB.1.19 cells in the CLDN2-RNAi group (*P *= 0.004) were significantly increased following CLDN2 silencing (Fig. 4d, e).

### Impact of silencing of afadin on Ras/Raf/MEK/ERK pathway activation

**Fig. 5:**
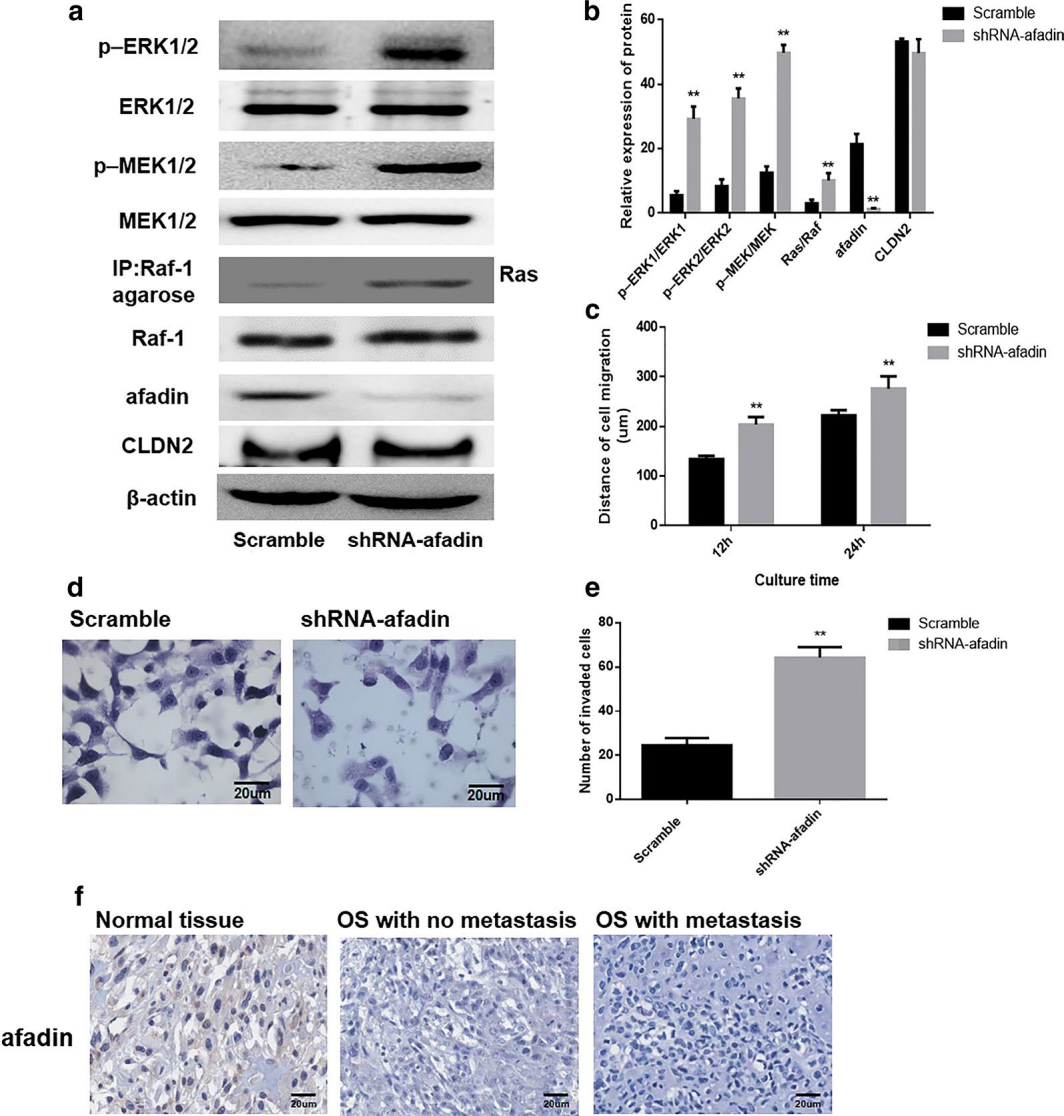
Impact of afadin silencing on the metastasis phenotype of U2OS cells. **a** Western blotting was used to examine the effects of silencing afadin and the activation of the Ras/Raf/MEK/ERK signaling pathway in the U2OS cell line. **b** The corresponding statistical analysis of the activation of the Ras/Raf/MEK/ERK signaling pathway. **c** The wound healing assay was utilized to detect the migration ability of the U2OS cell line in vitro. **d** The transwell chamber method was utilized to detect the impact of afadin silencing on the invasive ability of cells in vitro. **e** The corresponding statistical analysis of invaded cells. **f** Membrane expressions of afadin in OS tissues and normal bone tissues. **P < 0.01 compared with the scramble group

To determine whether CLDN2 affects the ERK signaling pathway by regulating afadin expression, a pGCSIL-scramble plasmid and a pGCSIL-afadin-RNAi plasmid were used to transfect CLDN2-expressing U2OS cells. Western blotting was used to analyze the effects of silencing afadin in these cells, and the results showed that compared with the control group, the expression of afadin was significantly reduced in afadin-RNAi group (Fig. 5a). Western blotting was used to examine changes in the activation state of ERK pathway. After afadin silencing, the phosphorylation levels of MEK1/2 (*P *= 0.0021; *P *= 0.0001) and ERK1/2 (*P *= 0.0023; *P *= 0.0011) were significantly increased in CLDN2-expressing U2OS cells (Fig. 5a, b). Besides, the loss of afadin have no notably impact on the CLDN2 expression (*P *= 0.796).

Futhermore, a transwell chamber assay and a wound-healing were used to analyze the effect of afadin on the metastasis ability of the examined cells. The results showed the migration distances of CLDN2-expressing cells at 12 h and 24 h were significantly increased following afadin silencing (*P *= 0.0021; *P *= 0.0013) (Fig. 5c). In addition, the numbers of invasive cells in the CLDN2-expressing cells were significantly increased following afadin silencing (Fig. 5d, e).

### Expressions of afadin in human OS

The expression of afadin was explored in the 27 specimens containing OS tissues and 10 specimens containing non-neoplastic bone tissue. The expressions of afadin located at cell membrane and cytoplasm were taken as positive. As shown in Fig. 5f, the expressions of afadin in OS tissues were remarkably lower than in non-neoplastic bone tissues (P = 0.0005).

The relationship among afadin expression and clinical pathological indicators was analyzed, and it was found that the expression of afadin was not associated with the age (*P *= 0.341), gender (*P *= 1.000), clinical staging (*P *= 0.584) or response to chemotherapy (*P *= 0.127) but associated with pulmonary metastasis (*P *= 0.0003) in OS patients (Table 2). Besides, Chi-square/Chi-square goodness of fit test showed that afadin was positively correlated with CLDN2 expression (φ = 0.793, *P *= 0.011) (Table 3).

**Table 2:** Expression of afadin and clinicopathological characteristics in OS patients

Item	N	Afadin (high)	Afadin (low)	*P*
Tumor tissue	27	11	16	< 0.001*
Normal	10	8	2	
Age (years)				
≤ 19	15	6	9	0.341
> 19	12	5	7	
Gender				
Male	16	6	10	1.000
Female	11	5	6	
Stage				
IA–IIA	10	4	6	0.584
IIB–III	17	7	10	
Response to chemotherapy				
Poor	9	3	6	0.127
Good	7	1	6	
NA (n = 8)	11			
Pulmonary metastasis				
+	21	7	14	< 0.001*
−	6	4	2	

*NA* not available

* Statistical significance was found with the Chi-square test/Chi-square goodness-of-fit test

**Table 3:** Correlation between the expression of afadin and CLDN2 in OS tissues

Item	CLDN2 (high)	CLDN2 (low)	*P*
n	9	18	
Afadin (high)	6	5	< 0.01*
Afadin (low)	3	13	

* *φ* Phi coefficient

## Discussion

Recent research has revealed that the expression of tight junction protein CLDNs is frequently altered in various cancers [23]. CLDN2 is one of the 27 members of the CLDN protein family, and the current understanding of the biological functions of CLDN2 is primarily limited to barrier protection, and cell connections [24]. Our research group found that CLDN2 was underexpressed in OS tissues, and we hypothesized that this decrease in gene expression may play a role in the metastasis phenotype of OS. To verify this hypothesis, we created an OS cell line stably expressing CLDN2 and an osteoblast cell line with a CLDN2 knockout. It is indicated that overexpression of CLDN2 significantly inhibited the metastasis and migration abilities of OS cells. Similar to our study, recent studies demonstrated that the CLDNs was frequently down-regulated in various cancers, for instance, the expression of CLDN1 was down-regulated in pancreatic cancer cells and that re-expression of CLDN1 reduced the invasive ability of these cells [23, 25]. By contrast, others have reported that the expression of certain CLDNs in tumors is associated with strong invasion and metastasis abilities [10, 26]. Thus, the various CLDNs may have different impacts on the biological behavior of a certain tumor [27–29]. One potential reason for this difference is that CLDNs may have specific functions in different cells and rely on different interacting molecules [30, 31]. For instance, CLDN1 was reported to induce cell migration and invasion through activation of the c-Abl-ERK signaling pathway in human liver cells [32]. Parallelly, it is revealed that CLDN18 coupled with EGFR/ERK signaling contributes to the malignant potential of bile duct cancer [33]. However, there have been few reports on the roles of CLDNs in OS, and the specific molecular mechanisms remain to be clarified.

Latest studies have shown that the cytoplasmic C-terminus of CLDNs contains a PDZ-binding sequence, which binds other tight junction proteins on the cytoplasmic side of cell membranes [34]. These intracellular proteins participate in cellular signal transduction and thereby regulate a series of cell behaviors [35]. In present study, afadin, which contains a PDZ domain ligand [36–39], was identified as a potential target gene of CLDN2 protein.

Our data showed that afadin silencing results in a reactivation of the ERK signaling pathway and promotes the metastasis phenotype in OS cells stably expressing CLDN2. Moreover, our data suggested that the expression levels of both CLDN2 and afadin are found to be associated with pulmonary metastasis in OS tissues, suggesting that reduced CLDN2 and afadin expression is likely to participate in the pulmonary metastasis of OS. Hence, the expression levels of afadin and CLDN2 have the potential to be useful as molecular markers for the diagnosis of OS as well as for the determination of prognosis.

## Conclusions

In the present study, we confirmed that CLDN2 significantly inhibits the migration ability of U2OS cells. We also performed an initial exploration of the molecular mechanism of this effect, finding that CLDN2 affected the Ras/Raf/MEK/ERK signaling pathway via afadin and ultimately decreased the migration ability of OS cells.

## Abbreviations

CLDNs: claudins
ERK: extracellular signal-regulated kinase
DMSO: dimethyl sulfoxide
MEK1/2: mitogen-activated protein kinase kinase 1/2
Ras: rat sarcoma
Raf: rapidly accelerated fibrosarcoma
TACC3: transforming acidic coiled-coil protein 3
TJs: tight junctions
SDS-PAGE: sodium dodecyl sulfate-polyacrylamide gel electrophoresis
ZOs: zonula occludens
